# Contrast Affects fMRI Activity in Middle Temporal Cortex Related to Center–Surround Interaction in Motion Perception

**DOI:** 10.3389/fpsyg.2016.00454

**Published:** 2016-03-30

**Authors:** Halide B. Turkozer, Zahide Pamir, Huseyin Boyaci

**Affiliations:** ^1^National Magnetic Resonance Research Center, Bilkent UniversityAnkara, Turkey; ^2^Department of Psychiatry, Marmara UniversityIstanbul, Turkey; ^3^Neuroscience Graduate Program, Bilkent UniversityAnkara, Turkey; ^4^Department of Psychology, Bilkent UniversityAnkara, Turkey; ^5^Department of Psychology, Justus Liebig University GiessenGiessen, Germany

**Keywords:** center–surround interaction, fMRI, motion perception, MT, spatial suppression, visual cortex

## Abstract

As the size of a high contrast drifting Gabor patch increases, perceiving its direction of motion becomes harder. However, the same behavioral effect is not observed for a low contrast Gabor patch. Neuronal mechanisms underlying this size–contrast interaction are not well understood. Here using psychophysical methods and functional magnetic resonance imaging (fMRI), we investigated the neural correlates of this behavioral effect. In the behavioral experiments, motion direction discrimination thresholds were assessed for drifting Gabor patches with different sizes and contrasts. Thresholds increased significantly as the size of the stimulus increased for high contrast (65%) but did not change for low contrast (2%) stimuli. In the fMRI experiment, cortical activity was recorded while observers viewed drifting Gabor patches with different contrasts and sizes. We found that the activity in middle temporal (MT) area increased with size at low contrast, but did not change at high contrast. Taken together, our results show that MT activity reflects the size–contrast interaction in motion perception.

## Introduction

As the size of a high contrast moving pattern increases, it becomes more difficult to judge its direction of motion. However, if the pattern has low contrast (e.g., below 5%) judging its direction of motion may become easier as its size increases. This “size–contrast" interaction in motion perception was first demonstrated by Tadin et al. (2003), and has been replicated several times since then (Tadin et al., 2006; Golomb et al., 2009; Glasser and Tadin, 2011; Foss-Feig et al., 2013; Melnick et al., 2013). Tadin et al. (2003) proposed that the effect is a consequence of center–surround antagonism, likely within the middle temporal (MT) cortex, a critical brain area for motion perception (Culham et al., 2001; Born and Bradley, 2005).

The “MT-hypothesis” was supported directly and indirectly with more recent findings. In a study that most directly support the MT-hypothesis, Pack et al. (2005) found that suppressive influences from surround were present for some neurons in macaque MT at high contrast, but weak or non-existent at low contrast. Tadin et al. (2011) showed that disrupting MT activity with transcranial magnetic stimulation (TMS) resulted in improved motion discrimination of contralaterally presented large, high-contrast moving stimuli. GABAergic interactions are often implicated with the surround suppression findings, which are supported by studies on special populations where size–contrast interaction is shown to be reduced (Betts et al., 2005; Tadin et al., 2006; Serrano-Pedraza et al., 2014). Yet, so far there has been no direct human neuroimaging study to show the correlates of the size–contrast interaction in area MT. In this study our goal is to directly test the MT-hypothesis using functional magnetic resonance imaging (fMRI).

To address this goal, we first investigated behavioral thresholds for detecting the direction of drifting sinusoidal gratings of varying size and contrast. Next, we studied the effects of stimulus size and contrast on the fMRI signal in the visual area MT. Our behavioral findings were largely consistent with the literature. Moreover, we found that the MT activity agrees with psychophysical findings. We discuss our results in a wider perspective of contrast mechanisms and surround interactions (e.g., Angelucci and Shushruth, 2013).

## Materials and Methods

### Participants

Five volunteers including the authors ZP and HB participated in the behavioral experiments (three female, ages ranged from 22 to 42). Participants were graduate students, researchers, and faculty members in National Magnetic Resonance Research Center (UMRAM), Bilkent University. A different participant (author HBT) took part in a pilot experiment to determine the optimal conditions for the main behavioral experiment. Six participants including the authors HBT and ZP (five female, ages ranged from 22 to 26) participated in the fMRI experiments. Three participants participated in both experiments. All participants reported normal or corrected-to-normal vision, and had no history for neurological or visual disorders. Ethics approval was granted by Bilkent University Ethics Committee for Research with Human Participants. All participants gave written informed consent in accordance with the Declaration of Helsinki, and were paid for their participation.

### Behavioral Experiments

#### Experimental Apparatus and Software

All experimental stimuli were generated in Java Programming Platform using our Psychophysics Programming with Java Package^1^ and presented on a CRT monitor (HP P1230, 22 inch, 1024 × 1280 resolution, 120 Hz). Presentation of correct luminance values was ensured by using a gray scale look-up table obtained through direct measurement of the luminance values (SpectroCAL, Cambridge Research Systems). Participants’ head was stabilized using a chin and head rest at a distance of 75 cm from the screen.

#### Stimuli, Task, and Analyses

In the behavioral experiment, we determined duration thresholds for discrimination of direction of motion of Gabor patches presented on a mid-gray background (22.99 cd/m^2^). The patches were vertical sine wave gratings (spatial frequency: two cycles per degree) weighted by two-dimensional isotropic Gaussian envelopes, whose sizes were defined as twice their standard deviations. The stimuli drifted to the left or to the right at a rate of 2°/s within the Gaussian envelope placed at the center of the screen. Direction of motion was determined randomly for each trial and was equally probable in either direction. For a more time-effective design some of the experimental parameters were determined using the results from the pilot experiments prior to the main experiment. Stimulus size varied across 1°, 2.5°, and 5.0° in diameter, and nominal stimulus contrast (amplitude of the sine wave grating) was either 2 or 65% (3 size × 2 contrast = 6 experimental conditions). Each condition was blocked in a separate session of 320 trials, and sessions were randomly ordered for each participant. On every trial, observers viewed a foveally presented drifting Gabor patch, and indicated the perceived direction of motion by a key press (two-alternative forced-choice). Auditory feedback was provided (auditory tone of 200 ms duration, 300 Hz for correct and 900 Hz for wrong answers). The duration of presentation changed adaptively from trial to trial, following two interleaved 3-up 1-down staircases. One staircase started from a very short duration, which made the task very hard, the other started from a long duration, which made the task relatively easier. There were 160 trials in each staircase. Discrimination thresholds (79%) for perceiving direction of motion were estimated using Palamedes toolbox (Prins and Kingdom, 2009) in Octave^2^. Discrimination thresholds and standard errors were calculated for each condition, averages for observers were computed. Analysis of variances (ANOVA) with two factors (size and contrast) was performed to compare the thresholds using SPSS Version 19 (SPSS Inc., Chicago, IL, USA).

### Functional MRI Experiments

#### MRI Data Acquisition

Scanning was performed on a 3 Tesla MR scanner (Siemens Trio) using a 12-channel phase-array head coil at the National Magnetic Resonance Research Center (UMRAM), Bilkent University. Each MR session started with an anatomical scan, followed by four runs of experimental scans, and an MT localization scan. Anatomical data were acquired using a high resolution T1-weighted 3D MPRAGE sequence (TE: 3.02 ms, TR: 2600 ms, flip angle: 8°, FOV read: 256, FOV phase: 224, spatial resolution: 1 mm × 1 mm × 1 mm). Functional data were acquired with an echo-planar imaging (EPI) sequence (TE: 35 ms, TR: 2000 ms, flip angle: 75°, FOV: 192 mm × 192 mm, in-plane resolution: 3 mm × 3 mm, slice thickness: 3 mm, number of slices: 28, slice orientation: parallel to calcarine sulcus). The first two volumes of the functional scans were discarded to allow for T1 saturation by the scanner.

#### Visual Stimulus Presentation Setup

Visual stimuli were back-projected using a video projector (NEC NP125 Projector, refresh rate 60 Hz) fitted with a long throw lens (NuView 489MCZ900 Projection Zoom Lens, Navitar) onto a translucent back-projection screen that was viewed by participants with the help of a mirror attached to the head coil and located above their eyes while lying supine inside the scanner. The total viewing distance was 75 cm. Gray scale look-up tables were prepared and used for correct luminance presentation.

#### Experimental Design

**FIGURE 1:**
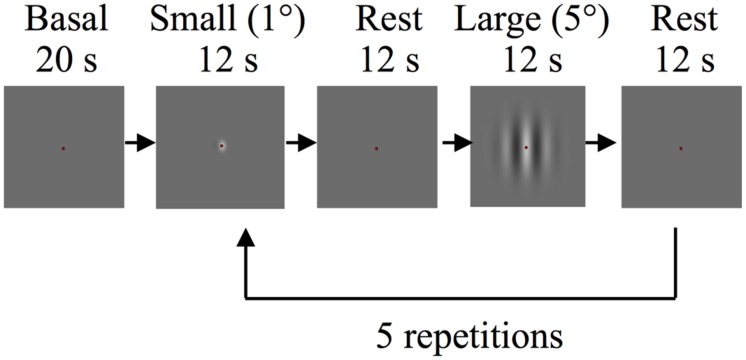
**Schematic depiction of the experimental procedure of the main functional magnetic resonance imaging (fMRI) experiment**.

In the main fMRI experiment, stimuli were drifting Gabor patches and were created using the same parameters as in the behavioral experiments. In each run subjects viewed five 12 s blocks of drifting Gabor patches at the center of the display (active blocks) alternating with 12 s of “blank” blocks (mean gray screen 22.99 cd/m^2^, baseline blocks). Each functional run lasted for 150 s including blank periods of 20 s at the start and 10 s at the end. During both types of blocks participants were required to maintain fixation at a central fixation mark. In each run 1° diameter (“small” condition) and 5° diameter (“large” condition) Gabor patches with identical contrasts were presented in alternating active blocks (**Figure 1**). The Gabor patches reversed motion direction every 2 s to minimize the effects of motion adaptation. Participants performed a demanding fixation task to minimize top-down modulation of the responses that are evoked by visual stimuli (e.g., see Moradi and Heeger, 2009). Participants were instructed to detect and report the color changes of the fixation mark using an MR-safe response box (Fiber Optic Response Devices Package 904, Current Designs). The fixation mark turned from its default color of red to blue or green randomly for 100 ms. Color changes occurred in every 750–1250 ms throughout the entire scan, including the blank blocks. In each session two experimental runs were conducted for different contrasts (2 and 65%).

#### MT ROI Localization

Regions of interest (ROIs) in area MT were identified independently through a separate run following the procedures developed in literature (Zeki et al., 1991; Tootell et al., 1995; Huk et al., 2002). The localizer run started with a 20 s blank period. This was followed by the following sequence of blocks. For 12 s a field of static white dots was presented on a gray background. The dots were randomly positioned within a circular region of 5° diameter centered at the fixation point. In the next 12 s block, of all the dots only those that were within the central 1° diameter were set in radial motion (alternating inward and outward every second). This was followed by a 12 s block in which only the peripheral dots, in the range from 1° through 5° diameter were set in motion. This sequence was repeated seven times in a run. Participants maintained fixation at the center of the stimulus throughout the entire run.

#### Data Preprocessing and Analyses

Magnetic resonance imaging data were preprocessed using BrainVoyager QX (Brain Innovation, Maastricht, the Netherlands). Functional preprocessing steps included 3D head motion correction, low- and high-pass temporal filtering, and slice scan time correction. The anatomical T1-weighted images were transformed into AC-PC coordinates. An inflated 3D model of the cortex is generated for each participant. The functional and anatomical images were aligned and functional maps were projected onto the inflated cortices for visualization per participant. ROIs were selected by a General Linear Model (GLM) procedure using Brain Voyager QX. In MT, areas that responded more strongly to central and peripheral moving dots were defined as inner and outer ROIs, respectively.

For each experimental run, time course of the fMRI signal was extracted from the independently defined ROIs for further analyses by our own numerical routines. The analyses included normalizing and computing the percent signal change with respect to the mean fMRI signal in that run. Event related averaging was then performed for each active block type, using the scan-averaged signal from the blank block as the baseline (the difference is computed between large and blank blocks, and between small and blank blocks at each time point). Next, we averaged those differences between 6 and 12 s (because the fMRI signal reaches its steady state plateau around 6 s). Paired sample Student’s *t*-tests were applied to the averaged data to determine the statistical significance. To further investigate the results we computed a spatial modulation index defined as ISM = *B*_Large_ – *B*_Small_, where *B*_Large_ and *B*_Small_ are the average responses to large and small Gabor patches, respectively. Mean ISM-values and standard deviations were calculated across the six participants.

## Results

### Behavioral Experiment

**FIGURE 2:**
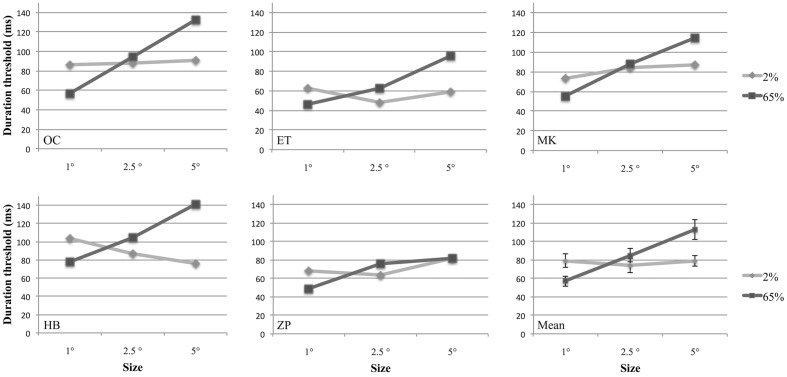
**Duration thresholds of five participants and the group means plotted as a function of stimulus size at 2 and 65% contrasts**. In the last figure (Mean), individual data points are averages of five participants. Error bars represent 1 SEM calculated across subjects.

In a behavioral experiment, we investigated how the size and contrast of a drifting Gabor patch affect observers’ sensitivity to discriminate its motion direction, and the interaction between these factors. For this purpose we estimated temporal discrimination thresholds for different sizes and contrasts for five participants using a two-alternative forced-choice design (**Figure 2**). A two-way repeated measures analysis of variance (ANOVA) with two factors (size and contrast) was performed to compare discrimination thresholds under different conditions. Contrast factor consisted of two levels (2 and 65%); and size factor contained three levels (1°, 2.5°, and 5° of visual angle). Results showed that there was a significant interaction between size and contrast [*F*(2,8) = 19.04, *p = 0*.009]. The main effect of size was also statistically significant [*F*(2,8) = 29.73, *p* = 0.002]. However, the main effect of contrast was not significant [*F*(1,4) = 29.73, *p* = 0.096].

Separate one-way ANOVAs were conducted for size at each contrast level. There was no effect of size at 2% contrast [*F*(2,8) = 0.41, *p* = 0.61], however, at 65% contrast, size affected the performance significantly [*F*(2,8) = 49.5, *p* = 0.0004]. Multiple comparisons of the data adjusted with Bonferroni correction showed that there was a significant difference between each pair of size level at high contrast (*p* = 0.001 for 1° versus 2.5°, and 1° versus 5°; *p* = 0.008 for 2.5° versus 5°).

Further analyses were conducted for the effect of contrast at each size level. For this purpose two-tailed paired-samples *t*-test was employed. To avoid family-wise error, Bonferroni Correction was applied and significance level was defined as 0.016 (0.05/3). We found that for small stimuli (1°), the average discrimination threshold was statistically significantly larger for low-contrast than for high contrast [*t*(4) = 9.3, *p* = 0.001]. Conversely, we found that the average discrimination threshold for high-contrast gratings was significantly higher for 2.5° [*t*(4) = –4.5, *p* = 0.014]. For 5° Gabors, although the discrimination threshold for high contrast was higher than low contrast, this difference could not reach statistical significance [*t*(4) = –3.2, *p* = 0.032].

#### Intermediate Discussion

Our results demonstrated that there was a significant interaction between the effects of size and contrast on motion discrimination. For small stimuli performance increased with increasing contrast; while for large stimuli, performance deteriorated with contrast. These results are consistent with previous findings in literature (e.g., Tadin et al., 2003). Although our behavioral results demonstrated decreased performance with increasing size at high contrast (spatial suppression), we did not observe a change in threshold with size at low contrast. This latter finding is different from the original findings of Tadin et al. (2003), who found a decrease in threshold with size at low contrast (“summation”). This disagreement will be discussed in more detail in the general “Discussion” section below. Nevertheless, because there is a systematic interaction between size and contrast, which is largely consistent with literature, it was sufficient to continue with the fMRI experiments for the purposes of our study.

### Functional MRI Experiment

To test the hypothesis that the patterns of activity in area MT correlate with the behavioral effect, we conducted an fMRI experiment. In a simple block design, we collected fMRI data while the participants viewed drifting Gabor patches with varying sizes and contrasts. We argue that, if an area is involved in processes leading to the behavioral results, it should exhibit systematically different patterns of activity for high- and low-contrast Gabor patches as their sizes vary, in a way that is consistent with center–surround antagonistic effects.

We set out to test the hypothesis in independently identified ROIs in the visual cortex. We aimed to identify two ROIs in MT: an “inner” ROI corresponding to the central 1° visual area, and an “outer” ROI corresponding to an annulus with an inner radius of 1° and an outer radius of 5°. However, those two localizers activated nearly identical tissue in MT. The tissue that was activated by the inner ROI localizer was always a subset of that activated by the outer ROI localizer, and repeated ANOVA results showed that there was no significant difference between the signal obtained in experimental runs extracted from the inner and outer ROIs [main effect of ROI: *F*(1,5) = 1.24, *p* = 0.32)]. This is not unexpected given the large receptive field sizes of MT neurons (Felleman and Kaas, 1984; Raiguel et al., 1995). Therefore, we do not present separate analyses from the two ROIs, instead we used the average of fMRI signals from inner and outer ROIs for further analyses.

**FIGURE 3:**
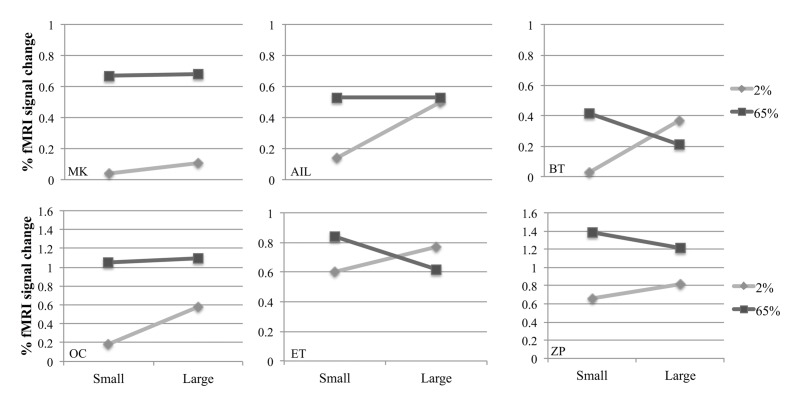
**Individual results from six participants in the fMRI experiment.** Points represent fMRI response amplitudes in the middle temporal (MT) regions of interest (ROIs) for small and large stimuli.

**FIGURE 4:**
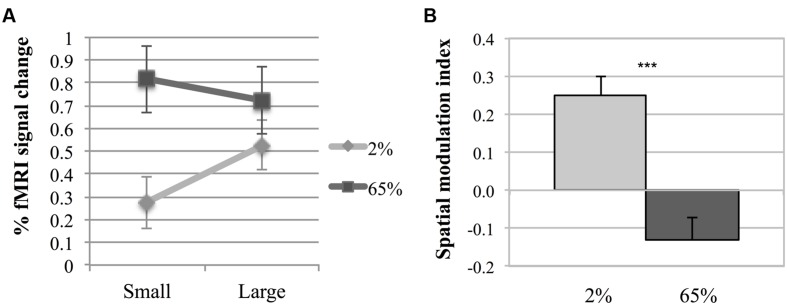
**Middle temporal activity. (A)** Two-way interaction plot of the impact of size and contrast on fMRI response amplitude in MT. Error bars are 1 SE calculated across subjects. **(B)** Spatial Modulation Indexes (ISM). ISM is defined as the difference between fMRI response amplitudes to large and small stimuli. Asterisks indicate a statistically significant difference between the ISM-values for low and high contrasts (***p ≤ 0.001). Error bars are 1 SE calculated across subjects.

**Figure 3** shows fMRI signal changes in MT for different experimental conditions for each participant. Different activation patterns were observed in MT in response to different contrasts as the size varied. A two-way repeated measures ANOVA was performed to investigate the effects of size and contrast on MT activity. Each factor had two levels (size factor: small, large; contrast factor: low, high). **Figure 4A** shows the average fMRI BOLD signal percent changes in different experimental conditions. ANOVA results demonstrated that, there was a significant interaction between size and contrast [*F*(1,5) = 29.22, *p* = 0.003]. While the contrast significantly affected MT activity [*F*(1,5) = 10.54, *p* = 0.023], the main effect of size was not significant [*F*(1,5) = 3.84, *p* = 0.11]. As the interaction between size and contrast was significant, we applied paired sample’s *t*-tests for multiple comparisons. Bonferroni correction was applied for multiple testing correction and the significance level was defined as 0.0125 (0.05/4). For small stimuli, the activity increased with increasing contrast [two-tailed paired sample *t*-test, *t*(5) = 5.51, *p* = 0.003]. However, for large stimuli, there wasn’t a significant difference between MT responses to low and high contrast [*t*(5) = 1.46, *p* = 0.2]. At low contrast, the response to large stimuli was significantly higher than that to small stimuli [*t*(5) = 4.67, *p* = 0.005]. On the other hand, at high contrast there was no significant change in activity as the size varied [*t*(5) = –1.88, *p* = 0.12]. These results show that MT activity increases with size at low contrast, while remains unchanged at high contrast.

To further investigate the fMRI effect, a spatial modulation index was computed (ISM = *B*_Large_ – *B*_Small_, where *B*_Large_ and *B*_Small_ are the mean responses to large and small Gabor patches, respectively, see “Materials and Methods” section). Positive index values reflect increase (“summation”), while negative values reflect decrease (“suppression”) in activity with increasing size. **Figure 4B** shows the index values. The spatial modulation index had a positive value for low contrast (*M* = 0.249, *SEM* = 0.051), and was statistically different than zero [one sample *t*-test, *t*(5) = 4.912, *p* = 0.004]. The index for high contrast had a negative value (*M* = –0.130, *SEM* = 0.060), but it was not statistically different than zero [*t*(5) = –2.15, *p* = 0.084]. There was a significant difference between ISMs for low and high contrast stimuli [paired sample’s *t*-test, *t*(5) = 5.44, *p* = 0.003]. These results demonstrate that although spatial summation was pronounced at low contrast, the effect of spatial summation on MT activity was not present at high contrast.

## Discussion

Under our experimental conditions, we found that the effect of size on motion direction sensitivity for drifting Gabor patches is dependent on stimulus contrast: performance increased with contrast for small stimuli, and decreased for large stimuli. Summarized alternatively, performance decreased with increasing size for high contrast stimuli, but did not vary for low contrast stimuli. This is partly consistent with previous findings by Tadin et al. (2003). In their work, they found decrease in motion direction sensitivity with size for high contrast Gabor patches as we found here. However, they also found increase in sensitivity for low contrast Gabor patches, whereas here we found no such increase. The discrepancy between the studies at low contrast could be because of differences in experimental conditions, and possible individual differences between the two groups of observers. For example, Tadin et al. (2003) applied Gaussian temporal ramps in their presentation, whereas here the stimuli appear and disappear on the screen with a temporal square-wave modulation. Using temporal ramps might have caused lower effective contrast compared to using square wave modulation as in our design. For this reason, the effective contrast of our stimuli might correspond to contrast levels that the transition from summation to suppression occurs [which was 5% contrast in the original study by Tadin et al. (2003)].

In the fMRI experiment, we found a significant interaction between the effects of size and contrast on MT activity. Moreover, the pattern of how spatial modulation indexes (ISMs) behaved under low and high contrast conditions was largely in line with the behavioral results: in the behavioral experiment, the signed difference between the sensitivities for small and large low contrast patches was higher than that for high contrast patches; consistent with this, in the fMRI experiment the ISM for low contrast was higher than that for high contrast. However, scrutinizing the results highlights subtle but systematic disagreements between the behavioral effect and MT activity: first, for high contrast Gabor patches motion direction sensitivity decreased with size but fMRI activity remained constant. Second, for low contrast Gabor patches the sensitivity remained constant but fMRI activity increased with size. However, we believe that these disagreements largely stem from non-linearities in the BOLD signal, and do not change the main conclusions. It is well known that there may not always be a linear relation between the BOLD signal and the stimulus energy. Stimulus energy may not drive neural activity linearly, and furthermore neural activity may not have a linear relation with BOLD signal (Logothetis and Wandell, 2004). Furthermore, our behavioral and functional paradigms have different characteristics such as different stimulus duration, stimulus direction or different experimental settings, which might affect neural responses as well as the BOLD signal.

Previous studies suggest that MT has much higher contrast sensitivity than in several other areas, and responses within MT tend to saturate quicker with contrast (Tootell et al., 1995; Buracas and Boynton, 2007) and field size (Durant and Furlan, 2014). Our results for large stimuli are congruent with these findings. At large stimuli size (5°), we found no change in MT activity with increasing contrast. On the other hand, for small stimuli, MT activity increased significantly as the contrast increased. This further supports previous suggestions that saturation of activity observed in MT depends on the size of the activated visual field (e.g., Tootell et al., 1995; Buracas and Boynton, 2007). Indeed, the saturation observed in MT may provide an alternative explanation for the discrepancy we found between the behavioral effect and fMRI activity in MT.

Even though our results support the MT-hypothesis, it is not possible to rule out likely contribution of other visual areas to the behavioral effect (for similar conclusions about the relation between motion perception and cortical activity see Thompson and Liu, 2006; Ciaramitaro and Boynton, 2007). Higher level visual areas such as medial superior temporal area (MST) and ventral intraparietal area (VIP) are involved in the analysis of visual motion and polymodal motion processing (Colby et al., 1993; Bremmer et al., 2001; Britten, 2008). Nelissen et al. (2003) showed that the motion response in VIP of macaque monkey significantly decreased with increasing stimulus size in an fMRI study. Cook and Maunsell (2002) found that the time course of the population responses of neurons in VIP but not in MT agreed with reaction time in a motion detection task. In the opposite direction of the information processing stream, feedback connections from MT to V1 enhance surround suppression in V1 (Hupé et al., 1998). Thus, the visual areas that receive projections from MT could be involved in the behavioral size–contrast interaction in motion processing. Further studies are needed to investigate the role of other visual areas.

The effect we find in area MT could as well be the product of input received from earlier visual areas, for example from V1. It is well established that the size of inhibitory and excitatory portions of the non-classical receptive field of a V1 neuron can be different for low- and high-contrast stimuli (“flexible receptive field”; Kapadia et al., 1999; Sceniak et al., 1999; Angelucci and Shushruth, 2013). However, there is no clear behavioral or neuroimaging evidence that suggests a similar mechanism in human under similar conditions (e.g., see Press et al., 2001; Zenger-Landolt and Heeger, 2003). Usually contrast sensitivity gets better with increase of the stimulus size independent of its contrast (e.g., see Meese and Summers, 2012; but also see Naito et al., 2014). Thus, it is critical to investigate V1 activity in relation to the behavioral effect. Unfortunately, we could not do so here because of the difficulty to draw boundaries between early visual areas (V1/2/3) in the so-called foveal confluence (Zeki, 1969; Dougherty et al., 2003).

Small eye movements can occur even under steady fixation conditions (Ratliff and Riggs, 1950; Martinez-Conde et al., 2004). Evidence in literature suggests that neurons in MT and MST play a critical role in eye movements (Kawano et al., 1994; Bair and O’keefe, 1998), and that eye movements affect their activity (Komatsu and Wurtz, 1988; Dukelow et al., 2001). On the other hand, oculomotor and perceptual processing of brief moving stimuli are shown to be independent from each other (Glasser and Tadin, 2014). These findings suggest that eye movements might have distinctive effects on behavioral results and fMRI activity. Although, we tried to minimize the effect of eye movements on neural response using a demanding fixation mark task during the fMRI experiment, eye-movements might have resulted in additive neural effects on MT activity.

GABAergic inhibitory interactions are often implicated with the surround suppression findings (Caldwell and Daw, 1978; Flores-Herr et al., 2001; Yoon et al., 2010; Thoreson and Mangel, 2012). Studies in special populations have yielded results supporting the role of GABAergic system. The behavioral effect was found to be reduced in populations exhibiting reduced cortical inhibition, including the elderly (Betts et al., 2005), patients with schizophrenia (Tadin et al., 2006) and with history of depression (Golomb et al., 2009). Reduced efficacy of the GABAergic system has been suggested to underlie the observed reduction in the size–contrast effect (Tadin et al., 2006; Golomb et al., 2009). Nevertheless, it is not clear which visual areas are affected by the reduced GABAergic transmission that results in behaviorally observed spatial suppression alterations in these clinical populations. Research on the processes involved in spatial suppression would provide a better understanding on how the brain, the visual system in particular, is organized. Furthermore, it would have broad clinical and practical impacts. Future neuroimaging studies on how psychiatric diseases affect spatial suppression would lead to a better understanding of both the pathophysiology of the diseases and the mechanism under spatial suppression. Further behavioral and neuroimaging studies are needed to verify the utility of spatial suppression as an endophenotypic marker of psychosis or major depressive disorder.

## Conclusion

Taken together our results suggest that, although the activity of MT is likely to contribute to the behavioral size–contrast effect in motion perception, it may not be the direct cause of the effect, and that other cortical processes could also involved. Future studies are needed to evaluate the function of other visual areas in size and contrast dependent center–surround interactions in motion perception.

## Author Contributions

HT, ZP, and HB made substantial contributions to the design, acquisition and analysis of the data, drafting the article and revising it. All authors gave final approval of the version to be submitted.

## Conflict of Interest Statement

The authors declare that the research was conducted in the absence of any commercial or financial relationships that could be construed as a potential conflict of interest.

## Footnotes

1 http://hboyaci.bilkent.edu.tr/PsychWithJava/index.html

2 http://www.octave.org
